# Is nitric oxide the forgotten nephroprotective treatment during cardiac surgery?

**DOI:** 10.1186/s13613-020-0631-7

**Published:** 2020-02-12

**Authors:** Mina Khorashadi, Michael P. Bokoch, Matthieu Legrand

**Affiliations:** 1 Department of Anesthesiology and Peri-Operative Medicine, University of California – UCSF Medical Center, 500 Parnassus Ave, San Francisco, CA 94143 USA; 2 Institute National de la Santé et de la Recherche Médicale (INSERM) 942, Lariboisière Hospital & F-CRIN INI-CRCT, Paris, France

Acute kidney injury (AKI) is a frequent complication in patients undergoing cardiac surgery with cardiopulmonary bypass (CPB), with a reported incidence of 30–50%, and an associated increase in mortality [1]. Proposed mechanisms of cardiac surgery-associated AKI include neurohormonal activation, inflammation, ischemia–reperfusion injury, hypoperfusion, venous congestion, microembolization and hemolysis. Nitric oxide (NO) is an important homeostatic mediator of renal hemodynamics [2]. NO deficiency occurs in various experimental models of renal injury, including during CPB. Data from animal studies suggest that abnormalities in endogenous NO production lead to kidney injury, due to an imbalance of production between the inducible and endothelial nitric oxide synthase [3].

The effect of NO therapy on AKI is controversial and may depend on the setting. In a randomized control trial including patients with multiple heart valve surgery (*n* = 244), NO delivered from the initiation of CPB reduced post-operative AKI and transition to stage 3 chronic kidney disease [4]. Contrary to these findings, a broadly inclusive meta-analysis (including studies of patients with sepsis and acute respiratory distress syndrome, ARDS, and surgical patients) showed no benefit of NO on post-operative renal function [5]. Of concern, the same study found that NO use was actually associated with worse renal function in patients with ARDS [6]. Given these conflicting results, how should the benefit of NO for prevention of AKI in cardiac surgery be adjudicated?

The meta-analysis by Hu et al. in this issue of Annals of Intensive Care addresses several of these discrepancies [7]. The authors analyzed five randomized controlled trials that investigate the effects of perioperative NO therapy on AKI after cardiac surgery. Notably, the included studies differ in two major parameters: the timing of NO administration and the definition of AKI. Of those analyzed, the two older studies initiated NO therapy immediately before weaning from CPB, and defined AKI as urine output less than 0.3 mL/kg/h or need for renal replacement therapy. These latter criteria likely underdiagnose the incidence of clinically significant AKI. In the three more recent studies (2018–2019), NO was initiated at the beginning of CPB and use the KDIGO criteria to define AKI. The pooled effect showed that perioperative NO therapy significantly reduced the risk of AKI after CPB with RR of 0.76 (95% CI 0.62–0.93, *p* = 0.008). Interestingly, NO therapy only showed benefit when initiated at the beginning of CPB (RR 0.71, 95% CI 0.54–0.94, *p* = 0.02). This result may imply that a key window exists where NO therapy can provide renal protection.

To interpret their findings, the authors emphasize the role of hemolysis in development of AKI after CPB (Fig. 1). Red blood cells are sheared as they go through the bypass circuit and release free hemoglobin (Hb) into the circulation. Free Hb in turn scavenges NO produced by endothelial cells, depleting plasma levels and ultimately resulting in vasoconstriction and reduced organ perfusion. Furthermore, free Hb and its degradation byproducts cause glomerular injury by forming reactive oxygen species, which can directly damage renal tubular cells and endothelium [3]. Thus, supplying exogenous NO in the peri-bypass period may not only protect organ perfusion by restoring the NO levels depleted by hemolysis, but also maybe by directly chelating free Hb and dampen its nephrotoxic effects. This is in contrast to ARDS where there is no apparent hemolysis. Moreover, prolonged NO therapy lasting days or even weeks (more commonly used in ARDS as compared with cardiac surgery) [6] has been associated with tubular apoptosis [8] and production of reactive nitrogen species leading to inflammation and renal injury.

**Fig. 1:**
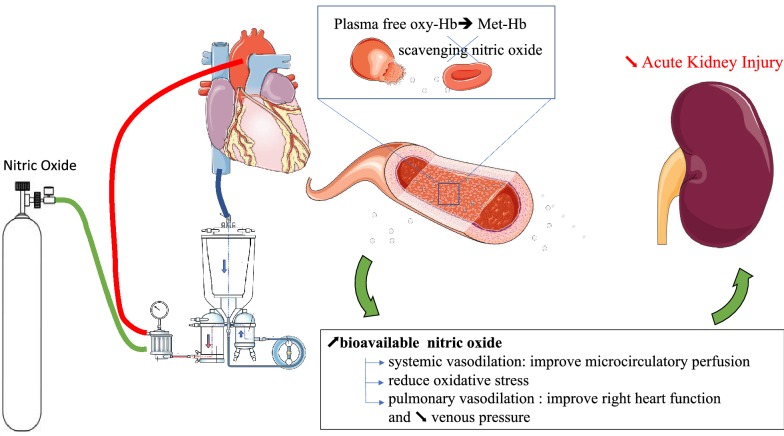
Proposed mechanisms of renal protection using nitric oxide during cardiac surgery with cardio-pulmonary bypass. *Free Oxy-Hb* oxyhemoglobin, *Met-Hb* methemoglobin

The conclusions drawn by Hu et al. encounter several challenges [7]. First, the small number of studies included in the analysis yield low statistical power. To provide additional statistical context for their findings, the authors performed trial sequential analysis, a method for adjusting significance thresholds in meta-analyses when the optimal sample size has not been reached. Given that the TSA monitoring boundary was not exceeded, the authors duly maintain that further studies are needed to confirm their basic finding. Second, the effect of NO on right ventricular function and venous congestion, both strongly associated with renal dysfunction [9, 10], are not explored in this meta-analysis. Given that right heart dysfunction is common after CPB, and that NO reduces right heart workload, the renal protective effects of NO may be mediated through improved right heart function. Unfortunately, of the studies included, only two reported hemodynamic data pertinent to right ventricular function, and the authors were unable to include this critical endpoint in their analysis. Finally, as discussed above, the finding that NO therapy lacks benefit when initiated only at the end of CPB is strongly confounded by the various definition of AKI used and the mode of administration of NO (i.e. inhaled or through the CPB circuit). This analysis is nevertheless important because it suggests that better delineation of the timing of NO therapy and its efficacy for preventing AKI warrants further investigation.

Limitations aside, what can clinicians learn from this information to help care for patients undergoing cardiac surgery? While no published trial to date has reported any adverse effects of NO during CPB, given the costs associated with NO therapy, its routine implementation for the sole indication of protecting the kidney is not practical in the absence of further data. The findings by Hu et al. may be limited by the small, heterogeneous pool of available literature, but they inform important new hypotheses about the timing and mechanisms of peri-bypass NO therapy. New studies inspired by this work are badly needed to better understand and manage AKI in this vulnerable patient population.

## Data Availability

Not applicable.
